# Healthcare Professionals Experience of Psychological Safety, Voice, and Silence

**DOI:** 10.3389/fpsyg.2021.626689

**Published:** 2021-02-19

**Authors:** Róisín O'Donovan, Aoife De Brún, Eilish McAuliffe

**Affiliations:** Interdisciplinary Research, Education and Innovation in Health Systems (IRIS), Health Sciences Centre, School of Nursing, Midwifery and Health Systems, University College Dublin, Dublin, Ireland

**Keywords:** healthcare, individual experience, teamwork, psychological safety, interviews (qualitative)

## Abstract

Healthcare professionals who feel psychologically safe believe it is safe to take interpersonal risks such as voicing concerns, asking questions and giving feedback. Psychological safety is a complex phenomenon which is influenced by organizational, team and individual level factors. However, it has primarily been assessed as a team-level phenomenon. This study focused on understanding healthcare professionals' individual experiences of psychological safety. We aim to gain a fuller understanding of the influence team leaders, interpersonal relationships and individual characteristics have on individuals' psychological safety and their decisions to engage in voice or silence behavior. Thirty-four interviews were conducted with healthcare professionals from across five teams working within an acute, suburban hospital. Hybrid inductive-deductive thematic analysis focused on identifying themes which captured the complexities of individuals' varied experiences of psychological safety. The themes identified were: “Personal Characteristics,” “Past Experiences,” “Individual Perceptions of Being Valued,” and “Judged Appropriateness of Issues/Concerns.” These themes are explored within the context of motivating and inhibiting factors associated with the influence of leadership, interpersonal relationships and individual characteristics on experiences of psychological safety and voice behavior. These results extend existing theoretical frameworks guiding our understanding of psychological safety by accounting for the variation in individuals' experiences and studying these significant influences on voice behavior. Important considerations for the development of interventions to enhance psychological safety are discussed.

## Introduction

Psychological safety concerns an individual's perception of whether it is safe to take interpersonal risks (Kahn, 1990; Edmondson, 1999). These interpersonal risks include engaging in open communication, voicing concerns, asking questions, and seeking feedback (Pearsall and Ellis, 2011). Psychological safety plays an important role in the workplace as it improves team learning (Edmondson, 1999, 2012; Rathert et al., 2009), creativity (Kessel et al., 2012; Lee, 2017), and performance (Singer and Edmondson, 2012; Edmondson and Lei, 2014; Newman et al., 2017). It plays a particularly important role in high stakes work environments, such as healthcare organizations, because it helps to ensure high quality care and patient safety (Nembhard and Edmondson, 2006). Psychological safety in healthcare teams has become increasingly relevant and important due to the ongoing COVID-19 pandemic. A strong, collaborative response to the pandemic requires creating psychological safety among healthcare professionals in order to enable them to collectively redesign processes and services to cope with new demands, learn from mistakes, integrate knowledge from healthcare organizations across the world and implement changes accordingly (Stoller, 2020). However, despite the importance of psychological safety for the effective operation of healthcare teams, there is an absence of evidence-based interventions which have explicitly targeted and improved psychological safety among healthcare professionals (O'Donovan and McAuliffe, 2020a). Past studies have demonstrated that educational and simulation-based interventions showed some improvements in psychological safety and/or speaking up behavior (Coyle et al., 2005; Dufresne, 2007; Pian-Smith et al., 2009; Johnson and Kimsey, 2012; Sayre et al., 2012; O'Connor et al., 2013; Raemer et al., 2016). However, many of these interventions have also shown mixed results or no significant change post intervention (O'Donovan and McAuliffe, 2020a). This previous research suggested that educational interventions alone may not be sufficient in order to change the deeply rooted behaviors associated with psychological safety and highlighted the need for multifaceted approaches to improve the effectiveness and efficacy of interventions targeting psychological safety in healthcare teams. A key part of developing effective interventions to improve psychological safety is to ground the design of the intervention in the experience of the end user (O'Donovan and McAuliffe, 2020a). Therefore, this study explores psychological safety through participants' experiences of engaging in voice or silence behaviors in their team. These lived experiences are complex and highly nuanced and thus, we adopt a qualitative approach to gain an in-depth understanding of lived experiences of psychological safety.

Past research has established the importance of psychological safety within healthcare teams. When healthcare professionals feel psychologically safe, they can engage in interpersonally risky behavior such as speaking up, sharing information, and seeking feedback (Nembhard and Edmondson, 2006; Pearsall and Ellis, 2011; Leroy et al., 2012; Bienefeld and Grote, 2014). These behaviors are integral to healthcare teams' ability to manage demanding conditions, constant changes in knowledge and practice, as well as their ability to learn from failure, adapt to new challenges, and make improvements (Nembhard and Edmondson, 2006; Carmeli and Sheaffer, 2008; Hirak et al., 2012). Conversely, when there is low psychological safety, people worry about taking interpersonal risks and engage in avoidance behaviors, such as silence.

However, research to date lacks an in-depth understanding of complex and nuanced experiences of psychological safety. While individuals who experience greater psychological safety are more likely to speak up (Edmondson and Lei, 2014), we cannot assume that employees who frequently speak up feel psychologically safe (Sherf et al., 2020) or that those who do not speak up are holding things back because they don't feel psychologically safe. Although employees' outward behavior, such as voice, may indicate that they feel psychologically safe, this may not reflect ideas, suggestions, and concerns that the individual is choosing to withhold. Acknowledging these nuances of psychological safety highlights the need to go beyond observable behaviors in understanding individuals' lived experience. By examining psychological safety in healthcare teams through a qualitative lens, our study will offer insights into the nuances and variation in healthcare professionals experiences of psychological safety.

Past research has identified some of the key factors which influence psychological safety and voice behavior. Morrison (2014) presents motivators and inhibitors of voice at the individual, organizational and interpersonal level, as well as contextual factors. Within a healthcare context, having familiar, trusting and supportive interpersonal relationships with a team leader and other team members fosters psychological safety (O'Donovan and McAuliffe, 2020b). When team members feel valued by one another it allows them to overcome the fear associated with taking interpersonal risks (Carmeli et al., 2009), making them feel safe to speak openly, learn and engage in their work (Kahn, 2007). However, psychological safety does not imply a team where people are necessarily close friends and where there is never any conflict or problems (Edmondson, 2003). In fact, psychological safety can facilitate the necessary “functional” and constructive conflict that is required to learn and improve how teams work together.

Previous studies have also noted that psychological safety is influenced by individual factors (Edmondson and Mogelof, 2006; Edmondson and Lei, 2014; Newman et al., 2017; Eibl et al., 2020; O'Donovan and McAuliffe, 2020b). A recent study conducted by Song et al. (2019) shifted focus away from extrinsic motivators of voice behavior to the role played by intrinsic motivators. Their study found that when individuals feel trusted by their team leader it facilitates a sense of psychological safety and, as a result, voice behavior. We aim to build on the knowledge generated by these studies to explore the nuance and variation in the ways these enablers of psychological safety are effective and ways in which they are not, i.e., when, where and how they make healthcare professionals feel psychologically safe. While the important role played by leaders and positive interpersonal relationships has been established, there is a need to examine the impact of leadership on psychological safety from multiple perspectives in order to develop a deeper understanding of how they influence team members decision to engage in voice. Roussin et al. (2016) suggest that although the team leader may be considered trustworthy and inspire psychological safety while team members are directly under their supervision, the absence of this leader may cause team members to revert to natural behavioral patterns. In this case, non-leaders or other team members may be influential in shaping psychological safety. Therefore, there is a need to further examine the extent to which both leaders and fellow team members influence individual team members' perceptions of psychological safety. This study examines participants' perceptions of both their team leader and their colleagues and the complex ways in which they influence their sense of psychological safety.

According to Morrison (2014), further research is necessary to expand our understanding of motivators and inhibitors of voice. Research at the individual level is needed in order to understand the ways in which employees' emotions and implicit beliefs may be influencing their decision to engage in voice behavior. Morrison (2014) includes many motivational variables associated with individual dispositions, however, there is only one inhibiting variable (achievement orientation) listed. This limited information on inhibiting influences warrants further investigation. The current study will extend this work by exploring the influence of intrinsic motivators and individual beliefs on healthcare professionals' decision to engage in voice behavior. Similar to the approach taken by Roussin (2008), our research is guided by grounded rationality. According to grounded rationality, individuals' goals, perceptions, and experiences lead them to make different sense of the same observed entity, action or situation (Weick, 1975, 1995, 2005). This perspective can be used to better understand how and why individuals with different experiences may experience psychological safety in different ways (Roussin, 2008).

Despite evidence that team members often possess divergent perceptions of team states and processes (Mathieu et al., 2008), current team level definitions and measurement of psychological safety do not acknowledge or account for teams in which members do not share similar beliefs about psychological safety. This limits our understanding of the variation in perceptions of psychological safety within teams (Roussin et al., 2016). Roussin et al. (2016) acknowledge and demonstrate that intact team and sub-team psychological safety dynamics coexist, interact, and may often misalign. Building on this work, our analysis will explore individual healthcare professionals' experiences to identify and understand variation between individual team members perceptions of psychological safety.

A qualitative approach is used to improve our understanding of the complex influence previously identified enablers and inhibitors have on psychological safety by accounting for the nuances of individuals experiences. To date, research into psychological safety has been predominantly quantitative in nature and reviews of the literature have called for the use of qualitative methodologies in order to gain a holistic understanding of psychological safety (Frazier et al., 2017; Newman et al., 2017). In this study, we address the need to incorporate the unique contribution qualitative research can make into our understanding of psychological safety. A qualitative approach is particularly suited to understanding individuals' experiences of complex phenomena, such as psychological safety. It offers a means to identify and understand aspects of experiences which quantitative methods and positivist perspectives may miss (Rousseau, 1997; King, 2000; Lansisalmi et al., 2000). We explored divergent perceptions of psychological safety within teams as well as individually held emotions and beliefs surrounding psychological safety and voice. Using qualitative methods to improve our understanding of psychological safety will ultimately improve our ability to develop interventions to improve psychological safety that are grounded in healthcare professionals' experiences. We address the following research questions: What are individuals healthcare professionals' experiences of psychological safety? Specifically, how do these experiences relate to their individual dispositions, their team leader or their relations with co-workers? These questions are explored and discussed within the context of informing the development of interventions to improve psychological safety in healthcare teams.

## Materials and Methods

### Research Setting and Participants

A total of 34 participants took part in this study. These participants were healthcare professionals who were recruited from five teams working within an acute, suburban hospital in Ireland. These teams varied from uni-disciplinary to multi-disciplinary and included both clinical and managerial staff. A purposive sampling strategy was employed to capture an understanding of individuals' experiences across different grades (team leaders, senior team members or junior team member) and disciplines [Physiotherapy (*n* = 13), Management (*n* = 8), Speech and Language Therapy (*n* = 7), Nursing (*n* = 4), Physician (*n* = 1), Administration (*n* = 1)] (Devers and Frankel, 2000). In addition, snowball sampling was used by asking participants who had been recruited to identify other team members who may be willing to take part in an interview. The inclusion criteria were: participants should be a member of at least one case study team; and could be from any discipline or work at any level within the teams. The team leader was defined by their formal role of management or co-ordination of the team. In addition, team members confirmed that they viewed this individual as their team leader during the interviews. Similarly, the team members identified themselves as either junior or senior team members during the interview. The senior team members had a longer tenure on the team, compared to junior team members. Table 1 provides detailed information on the teams and the participants in this study. Data collection continued until no further participants who were willing to take part in an interview could be identified.

**Table 1:** Participant and team information.

**Participant information**	**Team A**	**Team B**	**Team C**	**Team D**	**Team E**
Team type	Unidisciplinary	Multidisciplinary	Unidisciplinary	Unidisciplinary	Multidisciplinary
Position	Junior: *n* = 4 Senior: *n* = 9	Junior: *n* = 0 Senior: *n* = 8	Junior: *n* = 0 Senior: *n* = 4	Junior: *n* = 3 Senior: *n* = 4	Junior: *n* = 0 Senior: *n* = 4
Gender	Female: 12 Male: 1	Female: 7 Male: 1	Female: 4 Male: 0	Female: 7 Male: 0	Female: 3 Male: 1
Total	13^a^	8^a^	4^a^	7	4^a^

a *One participant was part of both Team A and Team E and another participant was part of Team B and Team C. These participants spoke about their experiences on both of their teams and so are counted twice in this table*.

### Data Collection

Individual semi-structured interviews were conducted with each participant. This was compatible with the study's aim of understanding individual team members' experiences and perceptions of psychological safety. Data collection was conducted by the primary researcher, who had been studying psychological safety within the hospital for a period of 8 months prior to conducting the interviews but did not have a pre-existing working or personal relationship with participants. Interviews were conducted in a private room located within the case study hospital, at a time that suited the participant. They lasted between 12 and 56 min, with an average length of 28 min.

The interview schedule was developed based on literature related to psychological safety, voice behavior, learning behavior, interpersonal relationships, and conflict, and has been published in O'Donovan and McAuliffe (2020c). Drawing on the principles of the Critical Incident Technique outlined by Flanagan (1954), team members were encouraged to recall and share incidents which were relevant to their experience of psychological safety, such as speaking up and remaining silent (Gremler, 2004). To explore differences in team members and team leader experiences, the interview schedule was modular and could be altered based on whether a team leader or a team member was being interviewed. Some generic probes and follow up questions were included to allow the interviewer to seek clarity without introducing response bias (Chell, 2004).

### Data Analysis

#### Thematic Analysis

Hybrid inductive-deductive thematic analysis was used to analyse the interviews. This involved “identifying, analyzing, and reporting patterns (themes) within data” (Braun and Clarke, 2006, p.6). Themes were identified within individual interviews. These themes were then compared across individuals within the same teams to search for consistencies and inconsistencies between individuals' experiences. Lastly, themes identified within each team were compared across teams. Each theme captured an important aspect of individuals' experience of psychological safety and was further developed with reference to the psychological safety literature. The current study uses thematic analysis because it facilitates participatory research where participants are viewed as collaborators in producing qualitative synthesis which can inform policy development (Braun and Clarke, 2006). It is a theoretically flexible approach to qualitative analysis which allows the combination of inductive and deductive methods (Boyatzis, 1998; Braun and Clarke, 2006).

#### Coding Process

A hybrid inductive-deductive approach was used to identify themes in the data which captured an aspect of participants' experience of voice or silence and exploring their relevance in the context of psychological safety literature. The process of analysis was informed by the approach outlined by Braun and Clarke (2006), Boyatzis (1998), and Miles and Huberman (1994). Analysis was supported by using the data management programme NVivo 12 (QSR International, 2015). Data analysis began after the first interview had been conducted and continued throughout the data collection process. The early stages of analysis involved writing a brief description of each interview, including notes on any changes needed in future interviews and the researcher's reflections on the interview completed. The interview was then transcribed. During this stage, the researcher became more familiar with the data and further developed interview summaries by writing memos on emerging ideas or themes. This was done using the “memo” function in NVivo.

Each interview was then coded. Coding occurred at each “meaning unit” defined as a data segment which contains one idea or theme and is comprehensible outside its context (Boyatzis, 1998). One researcher (ROD) coded the complete dataset and another (EMcA) independently double coded 10% of the interviews (*n* = 3). “Crystallization” assumes the goal of using multiple researchers is not to provide a more valid, singular truth but to identify and explore different facets of the research question (Tracy, 2010). In this research, crystallization was used to challenge the researcher's perceptions and ideas regarding the data and to develop a more complex and in-depth understanding of the phenomenon of psychological safety. Memos were used to track the development of these codes. Coding was conducted across two rounds; the first round of codes were data driven and descriptive while the second round reviewed and refined these codes with guidance from the findings of a systematic review of enablers of psychological safety in healthcare teams (O'Donovan and McAuliffe, 2020b). Specifically, the categories of “support,” “familiarity,” “hierarchy,” and “individual differences,” which were identified within the systematic review of enablers of psychological safety, informed this deductive analysis. These categories were used to clarify and refine our initial inductive codes. The codes generated by both coders were compared and discussed. While there was variation in the specific labels given to codes, both coders agreed on the codes attributed to each piece of interview text. During this discussion, the coders re-examined the research questions and their understanding of which codes were most relevant to understanding psychological safety. Following discussion between both coders, each code was given a clear, concise and unambiguous label, a definition of its meaning and a description of inclusion and exclusion criteria (Boyatzis, 1998).

**Figure 1:**
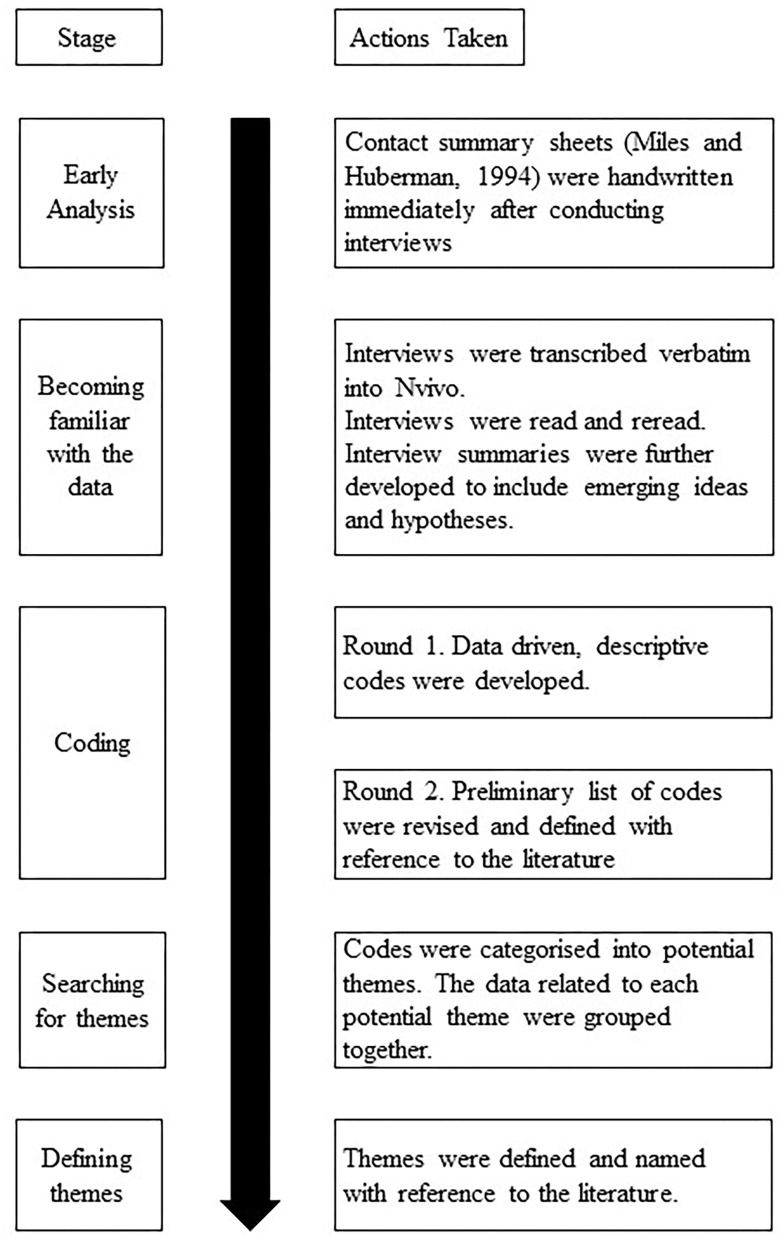
Overview of analysis process.

Codes were then categorized and grouped into themes. This was done by grouping similar codes and exploring other connections between codes and was completed within each team (within-case analysis) and across teams (cross-case analysis). Lastly, themes were defined and named. Each theme was reviewed with reference to the literature in order to aid clarity and ensure the relevance of each theme to the research questions. This was done concurrently with writing up the results of analysis. An overview of this analysis process can be seen in Figure 1.

### Reflexivity

The primary researcher engaged in reflexivity during the preliminary stages of this study and throughout data collection and analysis. In the preliminary stages, the researcher spent 8 months examining psychological safety within the hospital. This was an opportunity for the researcher to become familiar with the hospital culture and to identify appropriate teams to take part in the research. During this time, the researcher kept a reflective diary of her experiences. After each interview was conducted, a brief summary was written, and this included the researchers' reflection on the interview. During analysis, the “memo” function in Nvivo was used to record the researcher's reflection on the emerging codes and themes. Memos were also used to record summaries of the reflective discussions had by the research team during the analysis stage.

### Ethics

Ethical approval was obtained for this study from the Human Research Committee, University College Dublin. Written informed consent was obtained from all participants prior to commencing the interview. In order to de-identify interview transcripts, each one was assigned a code made up of P (participant), interview number (e.g., the first interview conducted within each team was given the number 1) and team letter (A, B, C, or D) and any identifiable characteristics were removed from the interview transcripts.

## Results

**Figure 2:**
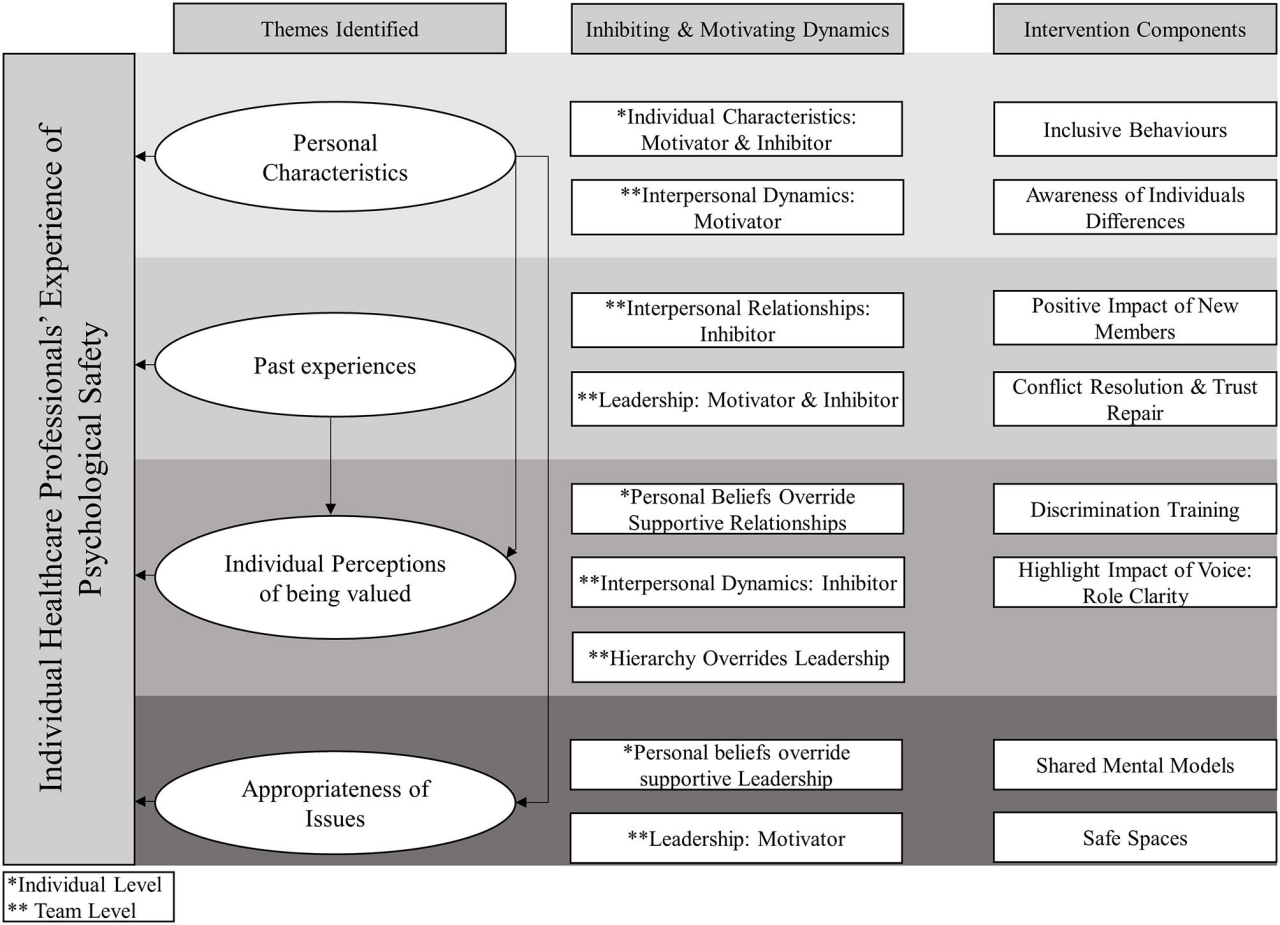
Overview of the experience of psychological safety themes, their associated inhibiting and motivating dynamics, and suggested intervention components.

Healthcare professionals' experiences of psychological safety are grouped according to the motivating and inhibiting effects that leadership, interpersonal relationships and individual characteristics have on voice and silence. These are discussed within four themes: “Personal Characteristics,” “Past Experiences,” “Individual Perceptions of Being Valued,” and “Judged Appropriateness of Issues/Concerns”. Figure 2 presents an overview of these results. Table 2 highlights the diversity of experiences within each team by indicating which themes appeared in each team. Theoretical saturation was deemed to be reached as each theme was identified from at least one participant within each team.

**Table 2:** Themes identified within each team.

**Teams**	**Personal characteristics**		**Past experiences**		**Perception of being valued**		**Perception of appropriateness of issues**	
**Impact on psychological safety**	**Positive**	**Negative**	**Positive**	**Negative**	**Positive**	**Negative**	**Positive**	**Negative**
Team A	✓	✓	✓	✓	✓	✓	✓	✓
Team B	✓	✓	✓	✓	✓	✓	✓	✓
Team C	✓	×	✓	✓	✓	✓	✓	✓
Team D	✓	✓	✓	✓	✓	✓	✓	✓
Team E	×	✓	✓	×	✓	✓	✓	×

✓ *indicates that the theme was brought up by members of this team*.

× *indicates that the theme was not brought up by members of this team*.

### Personal Characteristics

When asked about psychological safety, participants discussed the influence their personal characteristics had on their experiences. They did so in contrasting ways; there were participants who said that their personal tendencies made them feel comfortable engaging in voice behavior and others who said that their personal characteristics made them hold back. However, analysis also showed that interpersonal relationships and leadership played a role in the influence of personal characteristics on the decision to engage in voice behavior.

#### Individual Characteristics as Motivators of Voice

When referring to reasons for speaking up, P3C simply stated: “that's just the way I am.” Senior team members said that their direct communication style made it easy for them to engage in voice behavior. For example, a member of Team D identified herself as an outspoken person and said that other team members considered her to be “a straight talker” (P2D). She used this perception of her personal disposition as a justification for speaking up.

“I can almost use that to my advantage sometimes in meetings I might say, ‘look, I know I'm always the one who sees the problem here but like this might be what we'll run into I know the way you always say I'm a straight talker so I'm just going to say this”' (P2D).

#### Interpersonal Relationships as Motivator of Voice

In the example presented above, we can see that the influence of P2D's personal characteristics on her decision to speak up is not simply an intrinsic motivator. Her perception of her own personal characteristic is closely linked to her beliefs about how other team members view her. Her belief that other team members consider her to be a “straight talker” gives her reason to engage in voice behaviors.

#### Individual Characteristics as Inhibitor of Voice

Team members also attributed feelings of low psychological safety to their personal characteristics. P5D said that she would choose to remain silent because she was “quiet” or a “perfectionist,” which, she said, meant that she would not want to discuss a mistake she made with the team. Other participants said that their personal characteristics made them stay silent rather than risking engaging in conflict. Here we can see an exception to the influence of leaders on team members voice behavior. These team members clarified that their decision not to engage in conflict was not influenced by their team leader: “But that's me, that's nothing to do with {team leader} yeah, I, I'm one of those incredibly annoying people (laughs) who're just push overs.” (P6D). Similarly, although P2A thinks her team leader is open and supportive, she finds it difficult to challenge her. P2A says that she does not speak up about her perception of an unbalanced work distribution within the team because she wants to avoid initiating conflict. She attributes this tendency to her personality type, rather than the behavior of the team leader.

“I just think it's a personal, I'm not great with conflict anyway, and I do think that's a personal thing em, I don't think it's something, I think she'd take it well, and she'd be very open about it, I'm just not great at it myself.” (P2A)

##### Overcoming Inhibiting Effect of Individual Characteristics

Among team members who said that their personal characteristics made them more likely to remain silent, examples were also given of being able to overcome this. Although P2A and P2C described themselves as non-confrontational people, they would still speak up if they felt “passionate” about something. This suggests that the influence of individuals' personal characteristics on their decision to speak up or remain silent depends on their own personal feelings toward the issue at hand and can be overcome when the situation they are faced with compels them to speak up.

Team members made assumptions about the influence of personal characteristics on others' behavior. Participants said that certain team members were quiet or shy, and this could be the reason why they did not speak up: “that might be just their personality style and that might be the way they are with their friends.” (P1D). However, when exploring participants' perceptions of their fellow team members, it is important to note that perceptions of other team members' behavior may not be an accurate indicator of feelings of psychological safety. A member of Team D stated that while she can be viewed by other team members as being introverted and quiet, she feels comfortable within the team: “I might be, I can be perceived as being a little quiet at times or a little introverted em, but no it's definitely a team where I feel quite comfortable.” (P5D).

### Past Experiences

Participants reported that having positive past experiences made them more comfortable speaking up. These positive past experiences included speaking up about issues, feeling heard, and/or appreciated by other team members and having friendly or personal interactions with other team members. There were team members who indicated that they felt psychologically safe because they had been part of the team for a long time. Here, there was no reference made to whether or not their experience was positive or negative; simply working within the team for a long time was attributed to voice behavior and feeling psychologically safe. According to the team leaders, the most long-term and experienced members of Team B and Team E had the “biggest voices” (P2E), were more direct and would “say it as they see it” (P1B). Other team members said that their comfort speaking up stems from their experience of doing so: “yes I would, yeah, but I think that comes from experience you see and if you think it's really important, definitely I would.” (P3C).

#### Leadership as Motivator of Voice

Having a positive historical relationship with their team leaders was reported as a motivator of voice. While a member of Team A reported that she felt comfortable speaking up to the team leader because of her past experience working with her, she was aware that this level of comfort may not be experienced by more junior team members: “yeah it's easier for me, I'm sure it's probably harder for, em, for the youngers, because for them, that's the manager” (P4A). This suggests that having a historical relationship with the team leader flattens the hierarchy so that the leader is no longer seen as “the manager,” making it easier to speak up.

#### Leadership as Inhibitor of Voice

In contrast, past experiences could also have a negative impact on team members' psychological safety. Previous experiences of speaking up leading to no change or negative consequences, such as being embarrassed or ridiculed in front of others, caused team members to remain silent. A member of Team A felt she could not influence change and attributed this belief to her cumulative experience of working in the healthcare system.

“You kind of discover that after you work in a big organization like the {health care organization} for a few years, you just, you kind of, you try and challenge things em, but sometimes you just get a bit tired of it.” (P1A)

While the above example illustrates the influence of the larger systems context, a member of Team D shared her experience of speaking up behavior not being accepted or supported at the team level: “I realized pretty quickly that actually, this was a department that didn't really want to make big changes, and that I was seen as being a bit threatening, so I really backed off” (P2D). Early negative experiences of speaking up to the team leader and not being supported resulted in this team member feeling that her “voice is not allowed.” As a result, P2D was less likely to contribute “initiative and suggestions because of those early experiences.” P2D highlighted the key role played by the team leader in inhibiting her voice and, despite positive subsequent experiences, P2D still felt unsafe in her relationship with the team leader: “my guard I suppose is up, it's shaped by those early experiences.”

The experiences of the newer members of Team D contrast with P2D's early experiences. The newest team member felt supported and psychologically safe enough to explore and suggest new ideas: “I think there's a lot of like freedom to develop your own bit of work em, and yeah I think it works quite well” (P4D). P2D was aware that new team members did not have the “same impression” as her and acknowledged that the culture in recent years has shifted to become more “equitable” and inclusive. P2D also comments specifically that “management style is changing” and that the team leader is asking for more input from others. This suggests that the team leader has been able to positively influence the team culture in order to make new team members feel psychologically safe, but has not been successful in making those team members with a longer tenure feel the same level of safety. These contrasting experiences illustrate the diversity of experience within Team D, the extent to which P2D's historical relationship with the team leader impact her perception of psychological safety and may also indicate a changing culture within the team.

#### Interpersonal Relationships as Inhibitor of Voice

Historical interpersonal relationships played a key role in creating trust between members of Team B: “So it depends, I suppose, on the relationship that you'd have with people, you know, that you, you trust much more than, somebody that you'd trust much more, you're going to believe much more, even if they're talking bullshit.”(P6B). According to the interviews conducted with senior members of Team B, there was a history of conflict between members of Team B who had been part of the team for a long time. These historical team dynamics caused an undercurrent of a “lack of respect” (P2B) and a lack of trust due to past events. However, the lack of trust within the team was dissipated by the addition of new members. Similarly, a member of Team D commented that a new team member had encouraged more voice within their team through role modeling speaking up behavior:

“Just her way of being I think is going to be really positive for the department because I think that she's able to be a voice and do it in a way that comes across as not being confrontational.”(P2D)

According to P4B, new team members can be trusted because they have not yet demonstrated that they cannot be trusted: “maybe the trust is up because new team members haven't shown us that they can't be trusted” (P4B). This quote illustrates that P4B has a negative expectation of others and suggests that she expects new team members to eventually demonstrate their lack of trustworthiness. This negative framing may be based on past negative experiences as P4B mentions her trust in new team members in the context of her lack of trust in the other team members due to their past behaviors.

### Individual Perceptions of Being Valued

Team members' perception of whether their opinions would be valued impacted whether or not they engaged in voice behavior. These perceptions were linked back to their personalities, their past experiences, and their position/role in the team.

#### Position in Hierarchy Overrides Supportive Leadership

The hierarchical structure of teams inhibited the motivating role of inclusive and supportive leadership behavior. Despite team leaders being described by all participants as being inclusive, supportive, or approachable, some team members did not feel as valued within the team. Members of the same teams had different perceptions of being valued. While some members of Team A stated that not all team members were valued, others thought that “every team member is valued” (P9A). Some members of Team A and Team C said that they found it easy to speak up to their team leader because they believed that all team members were valued and trusted by the team leader: “she would always know {…} we all work really hard and support each other.” (P4A).

Those lower in the hierarchy reported feeling less valued and this inhibited their voice behavior. A member of Team A commented that, because of her junior position, there were times when she did not feel listened to or taken seriously by more senior colleagues.

“You know by the interaction the way it went you know that they aren't you know taking what you say on board, and possibly if you're kind of a younger member of staff or you look young or something like that, they might just be just like, ‘oh yeah, whatever like.” (P1A)

While P2E said that all team members input is equally listened to, she also said that she was aware that others do not share this perception and that some team members do not feel as valued as her: “I would say to some people, you need to say that at the team meeting, ‘oh I couldn't say that, oh no no no, will you say it? Because they'd listen to you but they wouldn't listen to me'.” (P2E).

#### Interpersonal Relationships as Inhibitors of Voice

Perceptions of not being valued may come down to a “personality clash” (P1B) or a lack of respect for another team member, based on previous experiences. A member of Team C stated that she remained silent in the past because she believed that others would not take her idea/opinion on board: “that a particular person, or, you know, em, it's it's, there's not much point, they're going to listen and they're going to do their own thing anyway.” (P3C). She claimed that this assumption was based on past experiences of other team members not listening to her.

#### Personal Beliefs Override Supportive Interpersonal Relationships

Within Team D, one team member did not feel as valued as others because they had been part of the team for longer than her.

“I feel almost like it would be a bit presumptuous of me to be making demands or, em, expecting that my voice be heard to the same level as everyone else's who have been here for years.” (P6D)

She explained that this belief comes from her, rather than the rest of the team who she says would never make her feel like her input was not valued: “that's not the impression that I would get from them, or from any other member of the team at all” (P6D). This suggests that team members' belief as to whether their opinion is valued can stem from personal beliefs or assumptions, rather than external, team level factors. It illustrates again the variety in the experiences and perceptions of psychological safety among individual team members.

### Judged Appropriateness of Issues/Concerns

Team members varied in their perception of whether issues were appropriate to raise at work. There were team members who said that they would only speak about personal or sensitive issues within small groups or with the team leader: “a number of things in my own personal life em, and {manager} has just been superb em with eh handling all of that, and eh, yeah I wouldn't have any eh concerns there at all” (P9A). However, others were reluctant to speak up about issues that they felt were of a personal or sensitive nature: “if it's more personal or something I really am struggling with, I'm not going to share with the team because it's just more of a personal preference” (P2A).

#### Leadership Acting as Motivator of Voice

Team E was described as having a familial atmosphere where it was easy to share personal issues with the team leader: “so when people come to me one to one, eh, over the years it's almost been exclusively personal, eh….and when I say exclusively, it's been 70% personal, 30% work.” According to interviews with members of Team B and Team D, group meetings were considered an inappropriate place to raise personal issues because they are meant to be formal business meetings: “it wouldn't be an appropriate thing, it's not a place for me to, that's a one-on-one” (P3B). However, they were happy to raise more personal issues with their team leader, in a one to one setting.

#### Personal Beliefs Override Supportive Leadership

Team members talked about being silent in relation to personal issues because they perceived the need for boundaries between the professional and the personal. P2D described feeling more uncomfortable raising personal issues, compared to work-related issues because they are less “black and white.” In addition, P2D said that she had remained silent about issues related to inequality in the team because it could have been interpreted as “being personal.” Due to her perceptions of professional boundaries at work, P4A reported being reluctant to speak up to her team leader about her personal needs. Although she described the team leader as being “extremely, extremely supportive,” she said that she is still reluctant to raise issues that impact her personally. This suggests that in this context, P4A's reluctance to speak up is due to her own judgement that it is not appropriate to bring personal issues into the workplace rather than her perception of how supportive her team leader would be. Another member of Team A said that she was reluctant to speak up to the team leader about personal issues. This team member described a culture in the team where all the other team members are “hard workers” and “high achievers.” This made her feel uncomfortable sharing things she was struggling with. Again, she attributed this belief to herself rather than team level factors: “But that's a personal issue, that's not a department problem.” (P6A). This illustrates that, similar to team members' beliefs about being valued, their belief about whether it is appropriate to raise personal or sensitive issues is an individual level belief that may not be impacted by the wider team.

## Discussion

The results from this study provide insight into individual healthcare professionals experience of psychological safety. They highlight the ways in which leadership, interpersonal relationships, and individual characteristics influence voice behavior and psychological safety. Interviews with healthcare professionals allowed us to explore and understand the variation in individual healthcare professionals' experiences of engaging in voice or silence behavior. While research to date has defined psychological safety as a shared team-level phenomenon (Edmondson, 1999; Edmondson and Lei, 2014; Newman et al., 2017), the current study focused on the individual level and explored the ways in which team members can vary in their perceptions of psychological safety. The qualitative analysis conducted offers a unique understanding of the complexity and nuances of individuals' experience of psychological safety. These findings provide a useful road map for developing interventions to improve psychological safety, which are grounded in individual experiences of healthcare professionals.

Our findings address the call for the impact of leadership on psychological safety to be examined from multiple perspectives in order to deepen our understanding of when and where leadership matters. They provide us with a fuller picture of the role of leadership in healthcare professionals decision to engage in voice behavior and their experience of psychological safety. Examples were given of how leaders could promote a sense of psychological safety and encourage voice behavior through positive historical relationships which flattened the hierarchy and made team members feel comfortable speaking up. Similarly, in interviews conducted by Attree (2007) nurses reported that having trust and confidence in managers facilitated reporting concerns. In contrast, past experiences of feeling silenced or not listened to by the team leader made participants reluctant to engage in voice behaviors, even after the leader had become more supportive and inclusive. As a result, team leaders were successful in making new team members feel psychologically safe but were not able to make team members with a longer tenure and negative experiences within the team feel the same level of safety. Other team members highlighted that their feelings of low psychological safety stemmed from their personal characteristics or beliefs and were not related to their team leader. This illustrates an exception to the influence of leadership on team members perception of psychological safety.

While previous research has shown that senior team members are more likely to feel psychologically safe (O'Donovan and McAuliffe, 2020b), our results indicate that they may remain silent due to a belief that speaking up will not result in any change. This corresponds to studies showing that employees engage in voice behavior when they believe there is a potential for them to make a difference (Sherf et al., 2020) and that silence occurs due to a belief that nothing will be done to address concerns (Milliken et al., 2003; Attree, 2007; Detert and Treviño, 2010; Moore and McAuliffe, 2010, 2012). Healthcare professionals in this study explained that their past experiences of not being listened to had taught them that there is often no point speaking up. Interviews conducted in previous research also revealed that experience of speaking up not having it's desired outcome lead both doctors and nurses to believe that doing so is pointless (Attree, 2007; Schwappach and Gehring, 2014). This may be an example of learned helplessness which states that after repeated punishment or failure, individuals become passive and remain so even after the environment has changed to make success possible (Overmier and Seligman, 1967).

Interpersonal relationships facilitated voice and psychological safety through positive past experiences with other team members. Previous research found that team members feel more psychological safety when they are familiar with one another and more experienced in their roles (O'Donovan and McAuliffe, 2020b). However, our analysis revealed that senior team members attributed feelings of low psychological safety to negative past experiences. This highlights that negative past experiences can override the positive impact familiarity should have on psychological safety. This finding resonates with Edmondson and Lei's (2014) suggestion that while psychological safety takes time to build, it can be destroyed in an instant through a negative response to an act of vulnerability. Team members commented on the challenges associated with moving on from negative historical team dynamics and cultures when the same team members were still there. This may be due to poor conflict resolution, as indicated by participants who reported avoiding engaging in conflict. Our findings highlight that while historically negative relationships and poor conflict resolution can threaten psychological safety, team members may be able to trust new team members. Developing trusting relationships with new team members could repair psychological safety within the team. However, past experiences may also create negative expectations of being let down by new team members. This emphasizes the crucial role of building trusting relationships between team members in order to create and maintain psychological safety.

Participants attributed their experiences of psychological safety and their decision to engage in voice behavior to their individual characteristics. Studies have shown that employee voice is associated with internal motivational states, such as having a prosocial mindset and action-oriented personality traits (Tangirala et al., 2013; Morrison, 2014). Interviews conducted with registered nurses have also highlighted that the beliefs and attitudes they learn at home influence their attitude to speaking up (Garon, 2012). Interestingly, in the current study, the perception that personal characteristics motivate voice was found among senior team members. According to the literature, healthcare professionals with a higher status also have higher levels of psychological safety (O'Donovan and McAuliffe, 2020b). However, while senior team members acknowledged that junior team members would feel less psychologically safe due to lack of familiarity with others, they did not explicitly comment on the positive influence their own status had on their levels of psychological safety. While it has been established that having a higher status contributes to higher psychological safety, our results suggest that those in a senior position may be unaware of the influence their higher status has on allowing them to feel more psychologically safe. As a result, they may perceive that their comfort with speaking up is driven by personal characteristics. Alternatively, they may be less influenced or affected by external factors such as organizational culture and leadership behaviors, allowing their personal characteristics to play a stronger role. The influence of individual beliefs about being valued and whether issues were appropriate also illustrates the influence of individual characteristics. Interviews conducted by Schwappach and Gehring (2014) also identified a belief that speaking up may be inappropriate, however, this was specifically in the context of speaking up to superiors, rather than concerns related to the appropriateness of specific issues, which was identified in the current study. Beliefs are part of personality and underlie an important aspect of adaptive functioning and can be influenced by past experiences (Dweck, 2008; Priest and Seemiller, 2018). Morrison (2014) proposed that the strongest inhibiters of voice behavior are deeply rooted fears and implicit beliefs that can cause employees to rationalize and justify their choice to remain silent. Similarly, our findings illustrate that healthcare professionals attribute their beliefs about being valued and whether issues were appropriate to their silence behavior.

Participants also attributed other team members' behavior to their personal characteristics, rather than any external or situational influence. This could be due to fundamental attribution error which says that observers tend to overestimate personality or dispositional cases of behavior and underestimate the influence of situational constraints on behavior (Ross, 1977; Jones, 1979). Considering our findings through this lens, participants tend to perceive other team members' voice or silence behavior as being influenced by their personal characteristics rather than external or situational factors such as their lower position in the team's hierarchy or lack of support from other team members. Examples were also given of participants overcoming their individual tendency to remain silent when they felt an issue was important enough to do so.

### Implications for Practice and Future Research

Our findings can be used to inform the development of interventions to improve psychological safety in healthcare teams. Figure 2 presents these findings, along with specific recommendations for the development of interventions. Arrows are used to illustrate the causal relationships between the themes identified and healthcare professionals experience of psychological safety, as well as the relationships between the themes themselves and with the motivating and inhibiting dynamics associated with each theme. The connections between themes and possible intervention components are also depicted.

A recent systematic review of team level interventions to improve psychological safety, speaking up and voice behavior within healthcare teams found mixed results for the effectiveness of such interventions (O'Donovan and McAuliffe, 2020a). Since psychological safety is a multi-level construct (Edmondson and Mogelof, 2006; Edmondson and Lei, 2014; Newman et al., 2017), there should also be a multi-level approach to improving it. Team leaders play an important role in creating and supporting psychological safety (Leroy et al., 2012) and this influence has been harnessed to improve psychological safety (Edmondson, 2018; O'Donovan and McAuliffe, 2020a). However, our results identified variation and exceptions to the influence of leadership on psychological safety and highlights the role all team members can play in creating and maintaining psychological safety. Leaders alone cannot improve psychological safety and there is a need for effort and involvement from all team members to create and maintain a safe environment. Interventions from outside the healthcare context have outlined the benefits of individual level interventions (Roussin, 2008) and the current study highlights the need for including individual level components in interventions targeting healthcare teams.

Table 3 presents intervention components which can inform the development of interventions to improve psychological safety in healthcare teams. In this table we outline three key focus areas for interventions, along with their associated components. These intervention components are described and linked to the categories proposed by Edmondson (2018) for introducing psychological safety. These include: setting the stage by reframing failure and clarifying the need for voice; inviting participation from all team members; and responding productively through expressing appreciation, destigmatizing failure and sanctioning clear violations when necessary. While Edmondson (2018) model is focused on the leader, the recommendations presented in Table 3 and described below, are aimed at all team members. Given the motivating effect positive interpersonal relationships have on psychological safety and voice behavior, interventions should focus on building trusting relationships. Suggested intervention components include conflict resolution, trust repair (Tomlinson and Mryer, 2009; Brown et al., 2011; Kim et al., 2013) and Improvisation Training (Maykowskyj Nordean, 2020). Overall, successful interventions, which draw on the other recommendations made in this paper, will improve psychological safety which, in turn will help team members reduce conflict and/or engage in “functional” task conflict which can result in learning and improved performance (Kostopoulos and Bozionelos, 2011; Bradley et al., 2012; Hoenderdos, 2013; Edmondson and Lei, 2014).

**Table 3:** Recommended components for to improve psychological safety in healthcare teams.

**Intervention focus**	**Intervention component**	**Description**	**Corresponding category for cultivating psychological safety (Edmondson, 2018)**
Building trusting relationships between team members	Conflict resolution and trust repair	Techniques to encourage respectful, open and direct communication between team members (Tomlinson and Mryer, 2009; Brown et al., 2011; Kim et al., 2013).	Setting the stage
	Improvisation training	Fosters psychological safety by increasing feelings of equality and encouraging team members to be present and listen to one another (Maykowskyj Nordean, 2020). The four main tenets of improvisation training are: Co-creation, accept, and heighten (“Yes and”), celebrating failure and listening (Leonard and Yorton, 2015).	Setting the stage
	Awareness of individual differences	Cultivate an understanding of the influence personal characteristics may have on team members experience of psychological safety and that this is something which can be overcome.	Setting the stage
	Inclusive behavior	Team members use words and deeds that invite and appreciate contributions from others.	Inviting participation
	Positive impact of new team members	Participants suggested that new team members can have a positive impact on trust and psychological safety within teams with negative historical relationships and cultures.	Inviting participation
Dealing with complex and/or sensitive issues	Foster shared mental models	Establish and agree upon the importance of sharing sensitive, personal, or complex issues that are impacting individuals' work.	Setting the stage
	Create safe spaces	Private spaces where team members can raise an issue that they find too difficult to raise with the whole team.	Inviting participation
Ensuring all team members feel valued	Discrimination training	Highlight the difference between prior and present situations, i.e., the value placed on each team member and the team's openness to hearing and acting upon all contributions. Team should develop ways to highlight how the issues being raised are being acted upon to make meaningful changes.	Responding productively
	Highlight impact of voice: role clarity	Focus on understanding roles and responsibilities in the team in order to establish the contribution each team member makes to ensuring the effective functioning of the team. The important contributions made by each team member should be openly shared within the team to ensure that members value one another and feel valued themselves.	Setting the stage

Interventions could also raise awareness of the influence personal characteristics may have on team members experience of psychological safety. Building on this awareness, interventions should offer mechanisms for encouraging voice behavior. Inclusive behaviors from other team members could be used to elicit opinions, encourage voice, and enhance psychological safety among individuals who feel their personal characteristics predispose them to have low psychological safety. Past research has indicated that inclusive leadership encourages team psychological safety (Nembhard and Edmondson, 2006; Hirak et al., 2012). However, our findings have highlighted that, although leaders play an important role, their behavior alone is not enough to create psychological safety for all team members. Therefore, this study calls on all team members to take responsibility for encouraging psychological safety by engaging in inclusive behaviors. Interventions to improve psychological safety should highlight that although team members may perceive their personal characteristics as inhibiting their voice, this is something which can be overcome when needed. Future research should explore the mechanisms through which individuals overcome their predisposition to remain silent and identify ways in which this can be promoted. According to Dweck (2008) interventions have successfully targeted beliefs, such as expectations of being accepted. This illustrates that it may be possible to overcome limiting beliefs that influence individuals' perceptions of psychological safety. Future research should focus on developing and implementing interventions which target individuals' perceptions of being valued within their team. Ensuring that all team members recognize their own value and contribution, along with the value of others, can improve psychological safety.

While O'Leary (2016) highlighted that having stable team membership can aid the development of psychological safety, participants in our study said that new team members can also have a positive impact on trust and psychological safety. They commented on the difficulty in overcoming negative historical relationships and culture without the introduction of new team members. Healthcare teams are vulnerable to high turnover rates (Kivimäki et al., 2007; Bong, 2019) and our results suggest some benefits from the addition of a new team member. Future research is needed to fully understand the positive impact new team members can have on psychological safety.

The complex and dynamic nature of healthcare settings means that healthcare professionals must feel comfortable negotiating and discussing a variety of complex and/or sensitive issues. Similar to conclusions made by Stühlinger et al. (2021), our findings highlight that, along with removing barriers to speaking up, we must address individually held attitudes toward speaking up. It is vital that interventions to improve psychological safety target individuals' attitudes toward dealing with sensitive issues. Table 3 presents two intervention components related to doing so. Teams should foster shared mental models by clearly establishing their expectations for what issues are important to raise, i.e., issues that are likely to impact on individual's performance and/or patient care. Participants uncertainty in relation to whether it was appropriate to raise complex, personal and/or sensitive issues within the team suggests a lack of shared understanding in relation which issues are important to raise. Shared mental models refer to team members overlapping mental representation of knowledge (Klimoski and Mohammed, 1994; Van den Bossche et al., 2011), in this case, the knowledge or understanding of which issues should be raised within the team. They have a positive effect on team performance, effectiveness and trust (Mathieu et al., 2000; Smith-Jentsch et al., 2005; Kellermanns et al., 2008; DeChurch and Mesmer-Magnus, 2010; Li et al., 2019), particularly in the context of complex tasks (Walsh et al., 1988; Van den Bossche et al., 2011). Developing shared mental models requires that the team share and agree on a mutual understanding of the meaning of the task, which acknowledges the complexity of the issue/task and incorporates the views of all team members (Tjosvold, 2008; Van den Bossche et al., 2011). It is also important that safe spaces are created where team members can raise an issue with another team member in private, when they feel it would be too difficult to raise it with the whole team.

Interventions should focus on ensuring team members know that their contribution will make a meaningful difference. Our findings revealed that team members often felt unsure about contributing their voice due to past experiences, even when their team had become more inclusive. Martinko and Gardner (1982) propose a model of Organizational Induced Helplessness which illustrates how environmental cues and individuals' historical experiences of outcomes influence their performance, i.e., their voice behavior. Building on suggestions made by Martinko and Gardner (1982), interventions could include discrimination training to highlighting the organization's openness to hearing their contributions and willingness to learn from their feedback. In addition, teams should focus on role clarity to ensure all team members are aware of the valuable contribution they make to effective team functioning, as well as the contributions made by others.

### Strengths and Limitations

This study builds on previous research by highlighting the individual differences which impact healthcare professionals' experience of psychological safety and engaging in voice behavior. By interviewing members of the same team, we were able to better understand that perceptions of psychological safety varied within teams and explore the leadership, interpersonal and individual level factors that influence this variation. However, some limitations must also be noted. Interviews were conducted with healthcare professionals within one case study hospital, restricting the generalisability of findings. We have addressed this limitation by providing a detailed description of the findings to allow readers to determine whether they are applicable in other settings (Gomm et al., 2000; Tracy, 2010). Although a diverse sample of professions and positions were recruited, the majority of participants were female and there was only one physician and one administrator interviewed. However, this reflects the gender balance within the teams and the number of teams which included physicians and administrative staff.

The primary researcher, who conducted all interviews, held an outsider position within the case study hospital: both as a post-graduate student primarily based in a university setting and as a non-healthcare professional. This may have shaped the researcher-researcher relationship and, as a result, the information that participants were willing to share (Berger, 2015). However, the researcher spent time within the case study hospital for 8 months prior to conducting the interviews, allowing time to become more familiar with the setting and to be recognized by participant as a researcher exploring psychological safety. In addition, all participants seemed comfortable during interviews and openly shared their varied experiences of psychological safety.

## Conclusions

Findings from this study highlight that there can be a wide variation in individuals' experiences of psychological safety within healthcare teams. Healthcare professionals attribute their experience of psychological safety to their personal characteristics, their past experiences, and their beliefs about whether they are valued and whether issues were appropriate ones for discussion within a team context. We explore these themes to extend our understanding of the influence leaders, interpersonal relationships and individual characteristics have on individuals experience of psychological safety. Our findings are discussed in the context of past research to further develop our understanding of psychological safety in healthcare teams and to inform future interventions to improve psychological safety.

## Data Availability Statement

The raw data supporting the conclusions of this article will be made available by the authors, without undue reservation.

## Ethics Statement

The studies involving human participants were reviewed and approved by Human Research Committee, University College Dublin. The patients/participants provided their written informed consent to participate in this study.

## Author Contributions

RO'D completed data collection and the first stage of data analysis. RO'D coded the complete dataset and EM independently double coded 10% of the interviews. All authors were involved in editing and finalizing the adapted measures. RO'D drafted the manuscript, AD and EM contributed to writing and revising the paper. All authors were involved in the design and planning of this study. All authors read and approved the final manuscript.

## Conflict of Interest

The authors declare that the research was conducted in the absence of any commercial or financial relationships that could be construed as a potential conflict of interest.
